# Inhibition of Chemokine (C-C Motif) Receptor 7 Sialylation Suppresses CCL19-Stimulated Proliferation, Invasion and Anti-Anoikis

**DOI:** 10.1371/journal.pone.0098823

**Published:** 2014-06-10

**Authors:** Mei-Lin Su, Tsung-Ming Chang, Chi-Hsiang Chiang, Han-Chen Chang, Ming-Feng Hou, Wen-Shan Li, Wen-Chun Hung

**Affiliations:** 1 Institute of Biomedical Sciences, National Sun Yat-Sen University, Kaohsiung, Taiwan, Republic of China; 2 National Institute of Cancer Research, National Health Research Institutes, Tainan, Taiwan, Republic of China; 3 Department of Surgery, College of Medicine, Kaohsiung Medical University, and Department of Surgery, Kaohsiung Municipal Ta-Tung Hospital, Kaohsiung, Taiwan, Republic of China; 4 Cancer Center, Kaohsiung Medical University Hospital, Kaohsiung, Taiwan, Republic of China; 5 Institute of Chemistry, Academia Sinica, Taipei, Taiwan, Republic of China; Taipei Medical University, Taiwan

## Abstract

Chemokine (C-C motif) receptor 7 (CCR7) is involved in lymph-node homing of naive and regulatory T cells and lymphatic metastasis of cancer cells. Sialic acids comprise a group of monosaccharide units that are added to the terminal position of the oligosaccharide chain of glycoproteins by sialyation. Recent studies suggest that aberrant sialylation of receptor proteins contributes to proliferation, motility, and drug resistance of cancer cells. In this study, we addressed whether CCR7 is a sialylated receptor protein and tried to elucidate the effect of sialylation in the regulation of signal transduction and biological function of CCR7. Our results demonstrated that α-2, 3-sialyltransferase which catalyze sialylation reaction *in vivo* was overexpressed in breast tumor tissues and cell lines. Lectin blot analysis clearly demonstrated that CCR7 receptor was sialyated in breast cancer cells. Chemokine (C-C motif) ligand 19 (CCL19), the cognate ligand for CCR7, induced the activation of extracellular signal-regulated kinase (ERK) and AKT signaling and increased the expression of cell cycle regulatory proteins and proliferation of breast cancer cells. When cells were pre-treated with a sialyltransferase inhibitor AL10 or sialidase, CCL19-induced cell growth was significantly suppressed. CCL19 also increased invasion and prevented anoikis by up-regulating pro-survival proteins Bcl-2 and Bcl-xL. Inhibition of sialylation by AL10 totally abolished these effects. Finally, we showed that AL10 inhibited tumorigenicity of breast cancer in experimental animals. Taken together, we demonstrate for the first time that CCR7 receptor is a sialylated protein and sialylation is important for the paracrine stimulation by its endogenous ligand CCL19. In addition, inhibition of aberrant sialylation of CCR7 suppresses proliferation and invasion and triggers anoikis in breast cancer cells. Targeting of sialylation enzymes may be a novel strategy for breast cancer treatment.

## Introduction

Many cellular protein undergo the post-translational modification called glycosylation in which glycans including monosaccharides, oligosaccharides and polysaccharides are conjugated to the asparagine (N-linked) or serine/threonine (O-linked) residue on the proteins [1]. The composition of the glycans of glycoproteins is diverse and various monosaccharides are added to the oligosaccharide chain via different covalent linkage [2]. Sialic acid, a monosaccharide with a nine-carbon backbone derived from neuraminic acid, is conjugated to the terminal position of oligosaccharides (a process known as sialylation) and is widely found on glycoproteins of eukaryotic cells [3]. Sialylation is a physiological reaction catalyzed by numerous sialyltransferases (STs) and is crucial for a variety of biological functions including cell adhesion, receptor activation, signal recognition and immune response [4], [5]. For instance, sialylation plays an important role in cellular interactions and can change the biological activity of immuoglobulins [6]. In addition, sialylation may modulate the function of dendritic cells by regulating antigen uptake, migration and T cell priming [7]. Moreover, a role of sialylation in the pathogenesis of neurodegenerative disorders like Alzheimer disease has been suggested [8]. Comparison of the serum ST activity showed a significant reduction in the Alzheimer disease patients [9]. Differences in sialylation of cerebrospinal fluid proteins was also found in the patients [10]. These results imply that alteration of protein sialylation may be involved in the development of a number of human diseases.

Aberrant sialylation has been shown to be a general phenomenon found in cancer cells and is strongly linked with proliferation, migration and invasion *in vitro* and *in vivo* [5], [11]. As examples, sialylation of epidermal growth factor receptor regulates receptor activity and increases chemosensitivity to gefitinib in colon cancer cells [12]. Sialylation of the Fas death receptor by ST6Gal-I provides protection against Fas-mediated apoptosis in colon carcinoma cells [13]. In addition, alteration of cell surface sialylation changes tumor cell adhesiveness and dramatically promotes metastatic potential [14], [15]. Hypersialylation of the glycoproteins of cancer cells could be regulated by (1) increased expression of enzymes that synthesize CMP-sialic acid, the substrate for sialylation, (2) up-regulation of STs that catalyze the addition of sialic acid to glycoproteins and (3) down-regulation of sialidases that cleave sialic acid during glycoprotein proteolysis. Our and others studies indicated that up-regulation of STs is frequently found in cancers and may be the major cause of cancer hypersialylation [15]–[17].

The interaction between chemokines and their cognate receptors is critical in tumor metastasis. An elegant study demonstrates that the chemokine receptors CXCR4 and CCR7 are highly expressed in human breast cancer cells, malignant breast tumours and metastases [18]. In addition, their respective ligands CXCL12 and CCL19/CCL21 exhibit peak levels of expression in organs representing the first destinations of breast cancer metastasis. Inhibition of the CXCL12/CXCR4 interaction by neutralizing antibody significantly impairs metastasis of breast cancer cells to regional lymph nodes and lung. Our previous study also showed that increase of the inflammatory enzyme cyclooxygenase-2 (COX-2) in breast cancer cells produced large amount of prostaglandin E2 (PGE_2_) to stimulate CCR7 expression and enhance lymph node metastasis of these cells [19], [20].

Whether chemokine receptors are sialylated *in vivo* has little been addressed. In addition, the role of sialylation on chemokine receptor signaling is also unclear. Until now, only two chemokine receptors CCR5 and CXCR2 have been demonstrated to be sialylated proteins. Bannert *et al* showed that CCR5 is O-glycosylated on serine 6 in the NH_2_ terminus and the sialic acid moieties contribute to binding of the chemokine ligands [21]. Frommhold *et al* demonstrated that sialylation by ST3Gal-IV significantly increased CXCR2-mediated leukocyte adhesion during inflammation *in vivo* [22].

We have previously developed a potent lithocholic acid-based and cell-permeable ST inhibitor AL10 which could suppress adhesion, migration and metastasis of lung cancer cells *in vitro* and *in vivo* by reducing sialylation of various integrin molecules [15]. In this study, we tried to study *in vivo* sialylation of CCR7 by using this inhibitor and explore the potential role of sialylation on CCR7-mediated signaling in breast cancer cells.

## Materials and Methods

### Breast Tumor Tissues

Ten paired normal adjacent and breast tumor tissues were surgically excised at the Department of Surgery, Chung-Ho Memorial Hospital, Kaohsiung Medical University. This study was approved by the Kaohsiung Medical University Chung-Ho Memorial Hospital Institutional Review Board. Written informed consent was obtained from all patients participated in this study.

### Cell Culture and Reagents

H104B5F5/M10 human mammary epithelial cells were purchased from Bioresource Collection and Research Center (Hsinchu, Taiwan) and were cultured in α-minimum essential medium containing 10% fetal calf serum (FCS) and antibiotics. MCF-7, MDA-MB-231 and SKBR-3 breast cancer cells were obtained from the same resource and were cultured in Dulbecco’s modified Eagle’s medium/F-12 medium containing 10% FCS and antibiotics. Synthesis of AL10 was described as previous study [15]. Sialidase was obtained from Takara Bio Inc. (Shiga, Japan). Human CCL19 and CCL21 recombinant proteins were purchased from R&D Systems (Minneapolis, MN). Anti-ST3Gal-I and anti-CCR7 antibodies were obtained from Epitomics, Inc. (Burlingame, CA). Anti-ERK, p-ERK, p38, p-p38, cyclin D1, cyclin A, and cyclin B antibodies were purchased from Cell Signaling (Danvers, MA). Anti-Caspase 3 antibody was obtained from Genetex (San Antonio, TX). Anti-Bcl-2, Bcl-xL, cyclin E and CDK1 antibodies were purchased from Santa Cruz Biotechnology (Santa Cruz, CA). Biotinylated Maackia amurensis lectin II was obtained from Vector Laboratories, Inc. (Burlingame, CA). Streptavidin–PE/Cy5 was purchased from Biolegend (San Diego, CA).

### Reverse-Transcription Polymerase Chain Reaction (RT-PCR)

Total RNA was isolated from tissues and cells and gene expression was investigated by using the one-step RT-PCR kit according to the manufacturer’s protocol. Glyceraldehyde-3-phosphate dehydrogenase (GAPDH) was used as an internal control to check the efficiency of cDNA synthesis and PCR amplification. The sequence of primers used are α-2,3-ST (for ST3Gal-I) forward: 5′-TCAGAGT GGTGCCTGGGAATGT-3′ and α-2,3-ST (for ST3Gal-I) reverse: 5′-TAGTGGTGCC.

AGTTCCCTTTGC-3′; GAPDH forward: 5′-AAGGCTGGGGCTCATTTGC-3′ and GAPDH reverse: 5′-GCTGATGATCTTGAGGCTGTTG-3′. CCR7 forward: 5′-GGACCTGGGGAAACCAAT-3′ and CCR7 reverse: 5′-GCCAGGTTGAGCAGG TAGGT-3′.

### Lectin Affinity Precipitation and Western Blotting

Control or AL10-treated cells were harvested at 24 h after treatment and cell lysate protein (1 mg) was incubated overnight at 4°C with 20 mg of biotinylated M. amurensis lectin II which could specifically bind to sialic acid in an α-2,3 linkage. Streptavidin-agarose beads were then added to pull down sialylated proteins, and samples were incubated for an additional 2 h at 4°C with rotation. Lectin/sialylated protein complexes were collected by centrifugation and washed twice with PBS containing 0.1% Triton-X 100. Sialylated proteins were released from the complexes by boiling in SDS–PAGE sample buffer. Proteins were resolved by SDS–PAGE as described previously [15] and probe with antibodies against CCR7 to detect the sialylation status of CCR7.

### Metylthiazoletetrazolium (MTT) Assay

Cells at the density of 8,000 cells per well was added into 96-well plates and treated with or without CCL19 for 24 or 48 h. After treatment, 100 µl of MTT (3-(4,5-dimethylthiazol-2-yl)-2, 5-diphenyltetrazolium bromide, 1 mg/mL) reagent was added to each well and incubated for another 4 h. After incubation, medium was removed and 100 µl of DMSO was added into each well. The absorbance at 570 nm was measured for evaluating cell proliferation.

### *In vitro* Invasion Assay

*In vitro* invasion assay was performed as described previously [19], [23] by using 24-well transwell units with poly-carbonate filters (pore size 8 µm) coated on the upper side with Matrigel (BD Biosciences). 5×10^3^ cells in 100 µl of medium were treated with 0.1% DMSO (vehicle) or 10 µM of AL10 for 24 h. Cells were harvested and placed onto the upper part of the Transwell unit. The lower part of the Transwell unit was filled with medium containing 0.1% BSA or CCL19 (200 ng/mL). After 24 h, non-invaded cells on the upper part of the membrane were removed with a cotton swab. Invaded cells on the bottom surface of the membrane were fixed in formaldehyde, stained with Giemsa solution and counted under a microscope.

### Anoikis Assay

To mimic the circulating cancer cells detaching from the surrounding extracellular matrix, cells were cultured in ultra-low attachment plate in complete medium containing 10 µM of AL10 or 200 ng/mL of CCL19. After 48 h, floating cells were collected by centrifugation and subjected for trypan blue exclusion assay to examine the viable cells. Cell lysates were also prepared for Western blot analysis to check the expression of pro-survival proteins and caspase-3 activation.

### Animal Study

Highly metastatic murine 4T1-Luc breast cancer cells (5×10^5^ cells/mice) were orthotopically injected into both mammary gland number four of female BALB/cByJNarl mice in a total volume of 100 µL. Mice were randomly sorted into two groups. The treatment was initiated with either the vehicle alone (Control) or AL10 (3 mg/kg/day intraperitoneally into each mouse every day). *In vivo* photon emissions of 4T1-Luc cells in mammary glands were detected and photographed by IVIS 200 image system at day 10. Photon flux reading from mice were averaged and expressed as Mean±S.D. The protocol of animal study was approved by the Institutional Animal Care and Use Committee of Academia Sinica.

### Statistical Analysis

Results are expressed as Mean±S.E. from three independent assays. Differences between two groups were examined by *t*-test and p<0.05 was considered statistical significance.

## Results

### Overexpression of ST3Gal-I in Breast Tumor Tissues and Cell Lines

**Figure 1:**
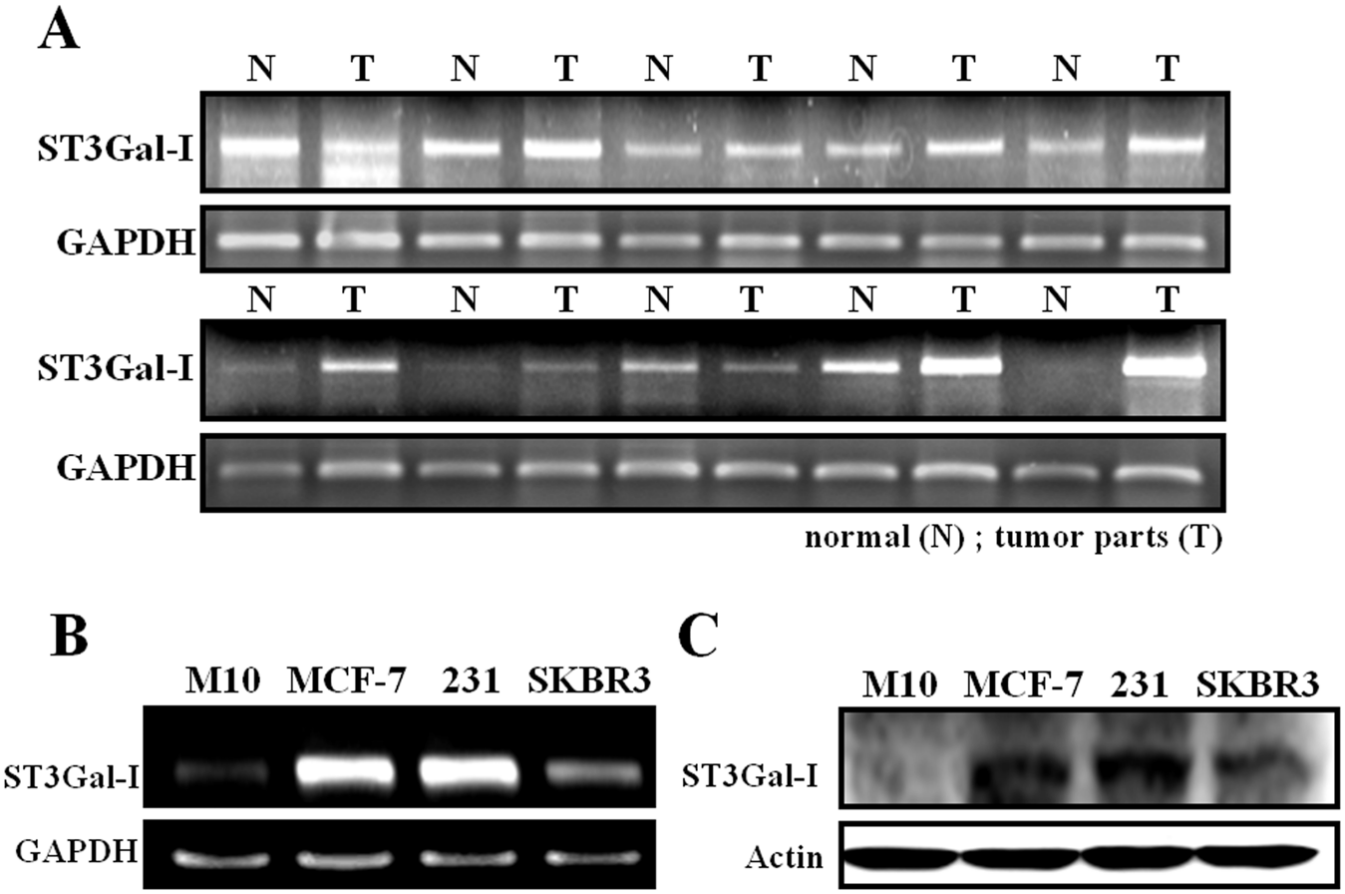
Overexpression of ST3Gal-I in breast cancer tissues and cells. (**A**) Total RNA was isolated from normal (N) and tumor parts (T) of breast tissues by TRIzol reagent and expression of ST3Gal-I was investigated by RT-PCR. (**B**) ST3Gal-I mRNA level was evaluated in M10 human epithelial cells, MCF-7, MDA-MB-231 and SKBR-3 breast cancer cells by using RT- PCR. (**C**) Protein level of ST3Gal-I was investigated by Western Blotting.

Two recent studies demonstrated that expression of the α2,3-sialic acid residues in breast cancer is associated with metastatic potential and overexpression of ST3Gal-I in transgenic mice promoted mammary tumorigenesis [24], [25]. Therefore, we checked the expression level of ST3Gal-I in ten breast tumor tissues and their adjacent normal parts by RT-PCR. We found that ST3Gal-I was overexpressed in 80% (8/10) of tested tumor tissues (Fig. 1A). Similarly, the mRNA and protein level of ST3Gal-I was also significantly increased in different breast cancer cell lines (including MCF-7, MDA-MB-231 and SKBR-3) compared to that of H104B5F5/M10 human mammary epithelial cells (Fig. 1B and 1C).

### Inhibition of CCR7 Sialylation Attenuated Downstream Signaling

**Figure 2:**
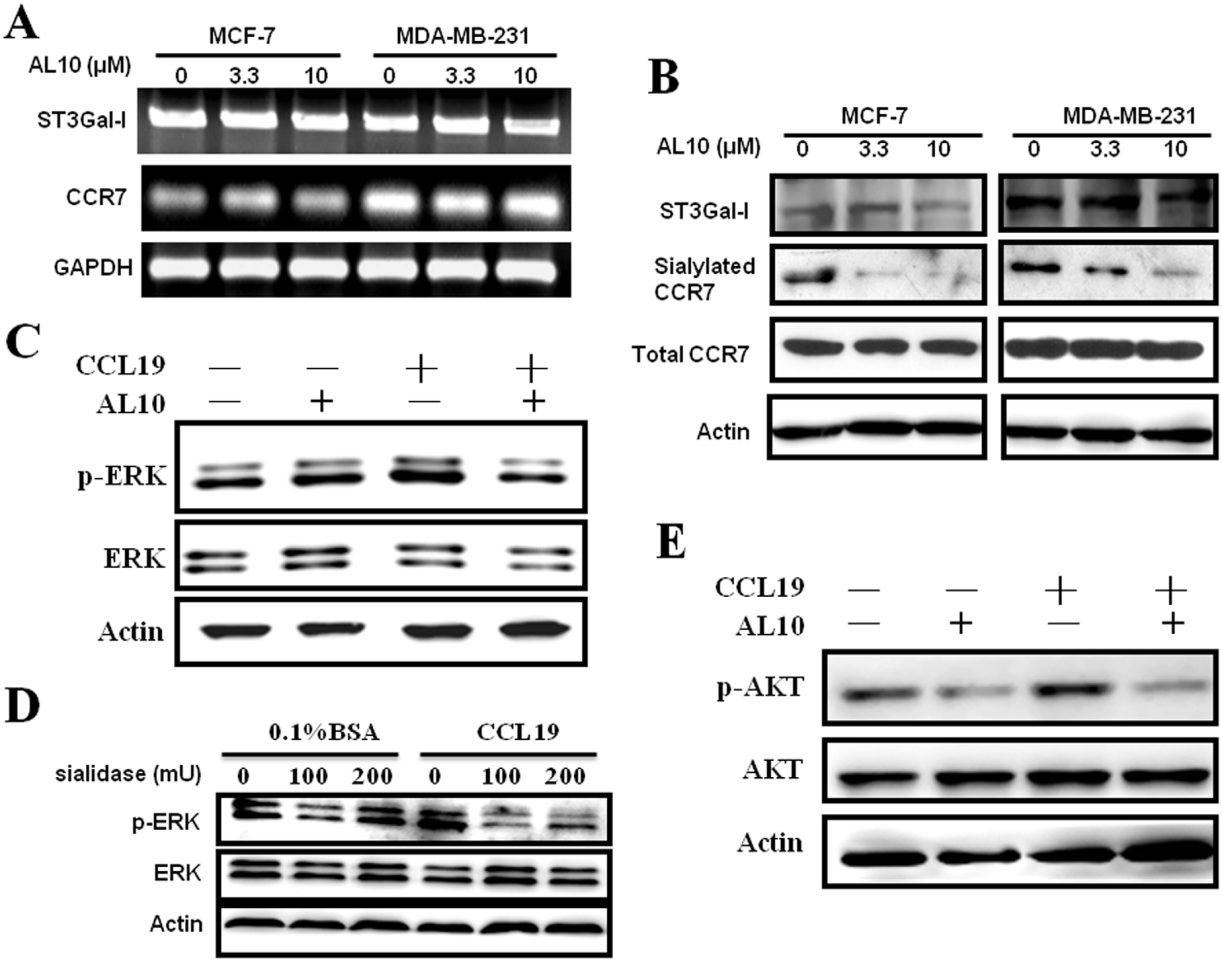
Inhibition of CCR7 sialylation attenuated its downstream signaling. (**A**) MCF-7 and MDA-MB-231 cells were treated with different dose of AL10 for 24 h and the expression of ST3Gal-I and CCR7 was analyzed by RT-PCR. (**B**) MCF-7 and MDA-MB-231 cells were pre-treated without or with AL10 for 24 h. A portion of cellular proteins was used to detect the expression of ST3Gal-I and CCR7 by Western blot analysis. Another portion of cellular proteins was incubated with biotinylated M.amurensis lectin II. Streptavidin–agarose beads were added to pull down sialylated proteins and the sialylation status of CCR7 was determined. (**C**) MDA-MB-231 cells were treated with AL10 (10 µM) for 24 h and then stimulated with CCL19 (200 ng/mL) for 20 min. Total ERK and phospho-ERK were detected by Western blot analysis. (**D**) MDA-MB-231 cell were pre-treated with different doses of sialidase for 3 h and then stimulated with CCL19 (200 ng/ml) for 20 min. Total ERK and phosphor-ERK were investigated. (**E**) Cells were treated as described in (C) and total AKT and phospho-AKT were detected by Western blot analysis.

We next addressed whether CCR7 is a sialylated protein. MCF-7 and MDA-MB-231 cells with high expression of ST3Gal-I were treated with different doses of AL10 for 24 h and the expression of ST3Gal-I and CCR7 was analyzed by RT-PCR. No significant change was detected in control and AL10-treated cells (Fig. 2A). In addition, AL10 did not affect CCR7 protein level (Fig. 2B). Interestingly, we found that CCR7 was sialylated *in vivo* as determined by lectin affinity precipitation and sialylation of CCR7 was significantly inhibited by AL10 in a dose-dependent manner (Fig. 2B). These data suggested that sialylation may play a role in the regulation of chemokine receptor function. To test our hypothesis, we studied whether inhibition of sialylation reduced CCR7 activation stimulated by its ligand CCL19. Extracellular signal-regulated protein kinase (ERK) and AKT are major downstream signaling molecules activated by CCR7 in different cell types [26]–[28] and are important for CCR7-induced prevention of apoptosis of human non-small cell lung cancer cells [27]. MDA-MB-231 cells were pre-treated with AL10 and then stimulated with CCL19 for 20 min. As shown in Fig. 2C, AL10 attenuated CCL19-stimulated ERK activation while it did not affect basal ERK activity. We also treated MDA-MB-231 cells with sialidase to remove sialic acid on cell surface of sialylated proteins and found that the activation of ERK by CCL19 was significantly inhibited by sialidase (Fig. 2D). CCL19 also activated AKT kinase activity which could be significantly inhibited by AL10 (Fig. 2E). Conversely, another CCR7 ligand did not significantly stimulate ERK and AKT activation under the similar experimental condition (Fig. S1) indicating the subtle differences in the regulation of CCR7 function by these two endogenous ligands. Therefore, we studied the effect of CCL19 in the subsequent investigations. Our data suggested that sialylation is important for CCR7 signaling.

### AL10 Inhibited CCL19-induced Cell Proliferation and Cyclin D1 Expression

**Figure 3:**
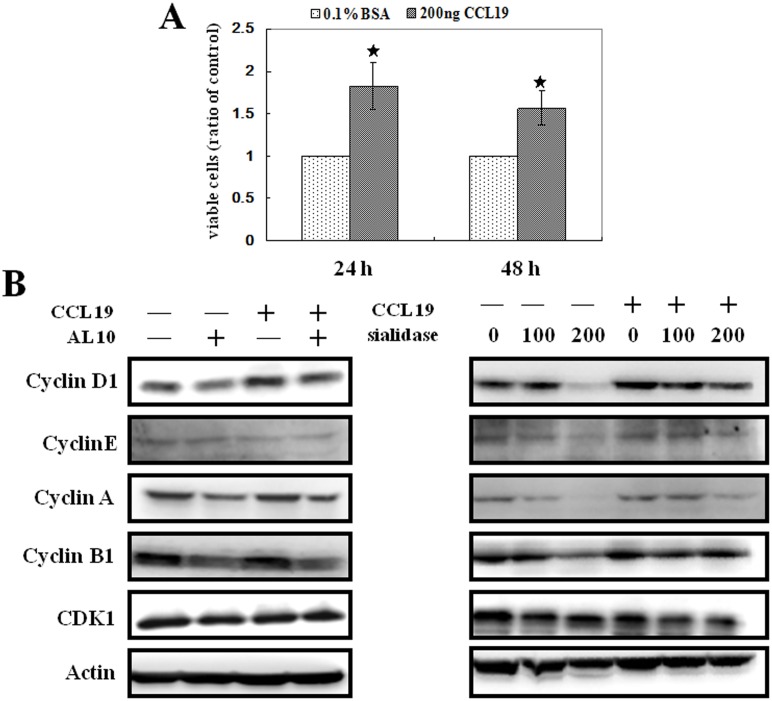
CCL19 stimulated cell proliferation and cyclin D1 expression. (**A**) MDA-MB-231 cells were incubated with CCL19 (200 ng/mL) for 24 or 48 h, MTT assay was performed to investigate cell proliferation, *p<0.05, n = 3. (**B**) MDA-MB-231 cells were pre-treated with AL10 (10 µM) or sialidase (100 or 200 mU) for 3 h and incubated with CCL19 (200 ng/mL) for another 48 h. Protein levels of cyclin A, B1, D1, E, and CDK1 were examined by Western blot analysis.

We next investigated whether inhibition of sialylation of CCR7 indeed repressed its biological function. Our results showed that CCL19 enhanced the proliferation of MDA-MB-231 cells (Fig. 3A). As shown in Fig. 3B, CCL19 increased significantly the protein level of cyclin D1 which could be inhibited by AL10. Interestingly, basal cyclin D1 protein level was also reduced by AL10 (Fig. 3B). Because cyclin D1 is critical for early G1 phase progression, inhibition of cyclin D1 also led to reduction of S and G2/M phase cyclins (including cyclin A and B1). However, the expression of cyclin-dependent kinase 1 (CDK1) was not affected. To verify this is a sialylation-dependent effect, we pre-treated cells with different doses of sialidase and then stimulated cells with CCL19. Our data demonstrated that CCL19-induced cyclin D1 was reduced by sialidase dose-dependently (Fig. 3B).

### CCL19 Increased Cyclin D1 Via a Post-transcriptional Mechanism Partly Via Glycogen Synthase Kinase-3β (GSK-3β)

**Figure 4:**
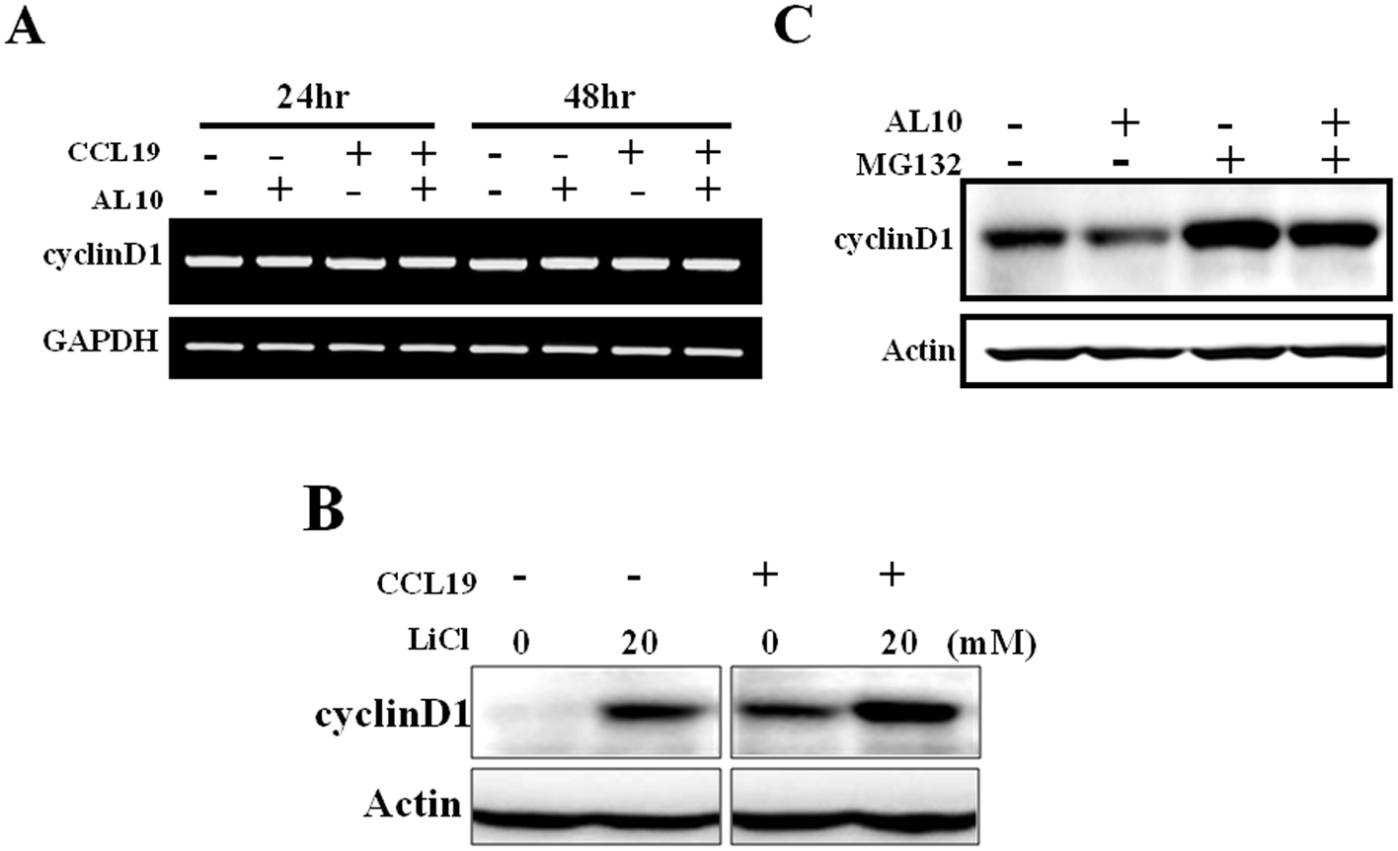
AL10 inhibited basal and CCL19-induced cyclin D1 expression via a post-transcriptional mechanism. (**A**) MDA-MB-231 cells were treated with different combinations of AL10 and CCL19 for 24 or 48 h. Cyclin D1 mRNA level was studied by RT-PCR. (**B**) MDA-MB-231 cells were pre-treated with LiCl (a GSK-3β inhibitor, 20 mM) for 3 h and then stimulated with CCL19 (200 ng/mL) for 48 h. Cyclin D1 protein level was analyzed by Western blot analysis. (**C**) MDA-MB-231 cells were treated with different combinations of AL10 and MG132 as described in Materials and Methods. Cyclin D1 protein level was analyzed by Western blot analysis.

We were interested in how CCL19 and AL10 modulated cyclin D1 expression because this oncoprotein is overexpressed in many cancers and is a potential target for cancer treatment. Addition of AL10 or CCL19 did not alter cyclin D1 mRNA level indicating a post-transcriptional regulation (Fig. 4A). GSK-3β has been shown to be a key regulator of cyclin D1 protein stability and is a downstream target of AKT. Because CCL19 activated AKT activity in MDA-MB-231 cells (Fig. 2E), we tested the potential role of GSK-3β in the regulation of cyclin D1 by CCL19. Under long-term serum starvation, cyclin D1 protein level was very low (Fig. 4B). Lithium chloride (LiCl), a GSK-3β inhibitor, increased cyclin D1 expression by suppressing protein degradation. CCL19 and LiCl showed partly additive effect on the accumulation of cyclin D1 indicating the overlapping of the signaling pathways modulated by these two agents and suggesting CCL19 may inhibit GSK-3β via AKT activation to up-regulate cyclin D1 protein (Fig. 4B). We also investigated the inhibitory effect of AL10 on basal cyclin D1 expression. When MDA-MB-231 cells were cultured under normal serum condition, cyclin D1 was constitutively expressed (Fig. 4C). AL10 alone reduced the protein level by about 50%. Addition of the proteasome inhibitor MG132 blocked cyclin D1 proteolysis and induced a 2–2.5 fold increase of protein level (Fig. 4C). AL10-induced reduction of cyclin D1 protein was completely reversed by co-treatment with MG132 suggesting AL10 down-regulated cyclin D1 via the ubiquitin-proteasome pathway (Fig. 4C).

### Inhibition of CCR7 Sialylation Reduced CCL19-induced Invasion

**Figure 5:**
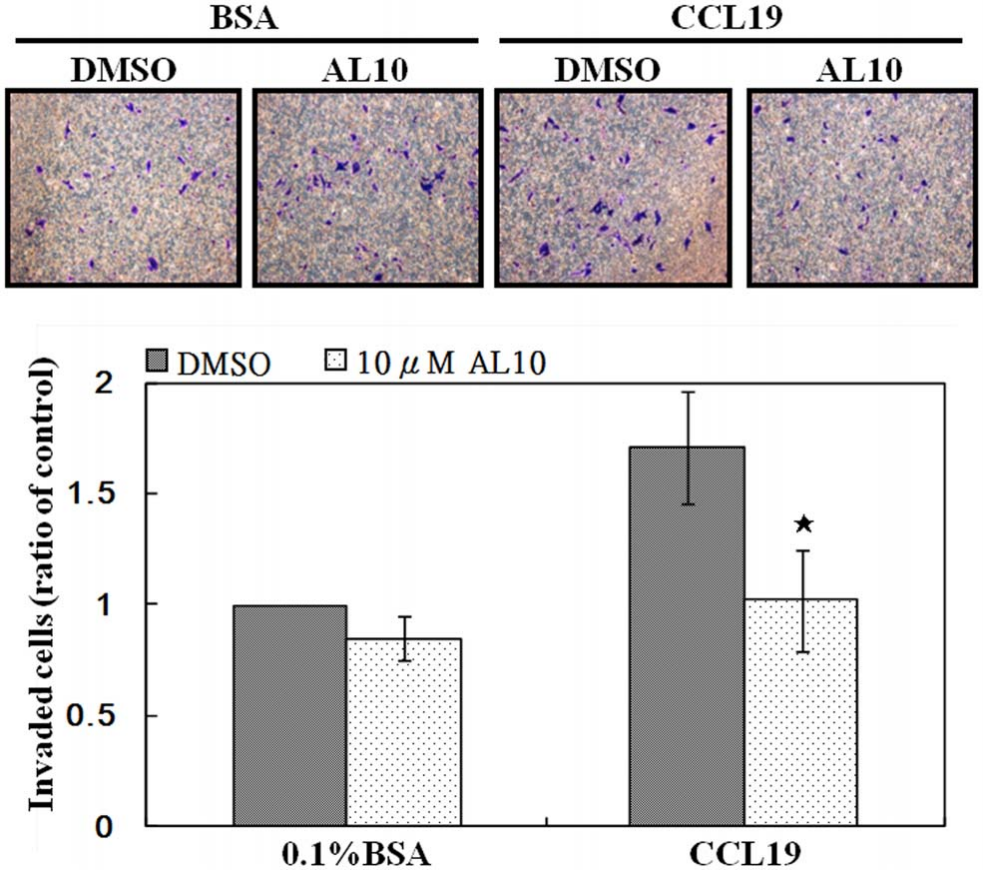
CCL19-induced breast cancer cells invasion was inhibited by AL10. MDA-MB-231 cells was treated with DMSO or 10 µM of AL10 for 24 h and then harvested for Transwell assay as described in Materials and Methods. Invaded cell number was counted. The number of invaded cells of the control group was defined as 1. *p<0.05, when compared to the control group. Representative images of invaded cells were shown.

We also found that CCL19 induced a chemotactic effect on CCR7-expressing MDA-MB-231 cells (Fig. 5). Pre-treatment of AL10 totally abolished CCL19-induced invasion of breast cancer cells (Fig. 5). However, the basal invasion ability of MDA-MD-231 cells was not affected by AL10 indicating the specificity of CCL19. This data also supported the notion that the CCL19/CCR7 signaling axis plays a role in the invasion of breast cancer cells into lymphatic vascular system (which expressed high level of CCL19) as demonstrated in our previous study [19].

### CCL19 Exhibited Anti-anoikis Effect on Breast Cancer Cells

**Figure 6:**
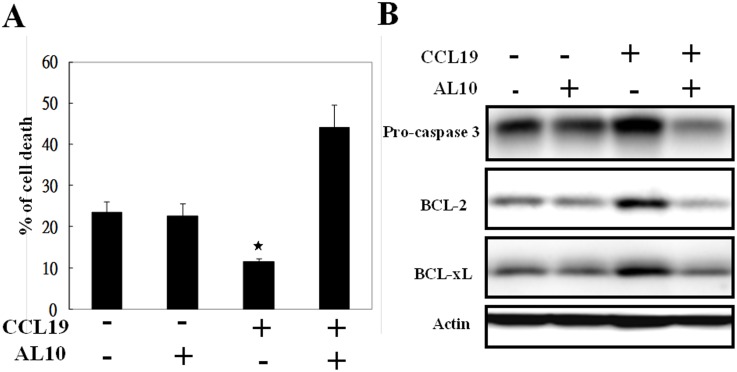
CCL19-induced anti-anoikis effect was abolished by AL10. (**A**) MDA-MB-231 cells were cultured in nonadherent attachment plate in the presence or absence of AL10 or CCL19 (200 ng/mL) for 48 h. Floating cells were collected to examine cell survive by trypan blue exclusion assay and the percentage of dead cells was expressed as Mean±S.E. from three independent assays. *p<0.05, when compared to the control group without CCL19 and AL10. (**B**) Expression of pro-caspase-3, Bcl-2 and Bcl-xL was investigated by Western blot analysis.

Anoikis is a kind of programmed cell death induced by lack of correct cell/ECM attachment. Metastatic tumor cells may escape from anoikis in blood or lymphatic system and colonize to distant organs. Pro-survival factors like Bcl-2 and Bcl-xl have been suggested to play an important role in the regulation of anoikis. To mimic the ECM detachment situation, MDA-MB-231 cells were grown in nonadherent condition for study. Under normal attachment culture, less than 2% of MDA-MB-231 cells underwent apoptosis (data not shown). The percentage of dead cells increased to 20–25% after culture under nonadherent condition for 48 h (Fig. 6A). CCL19 at the dose of 200 ng/mL caused about 50% of reduction of cell death (Fig. 6A) and significantly up-regulated BCL-2 and BCL-XL expression (Fig. 6B). Activation of caspase-3 was also suppressed as evidenced by increase of pro-caspase-3 in CCL19-incubated cells. Pre-treatment of AL10 totally diminished CCL19-induced protection from detachment-triggered cell death which was accompanied with caspase-3 activation and reduction of BCL-2 and BCL-xL (Fig. 6B). These data suggested that CCL19-induced anti-anoikis effect depends on CCR7 sialylation.

### AL10 Inhibited Tumorigenicity of Breast Cancer Cells *in Vivo*

**Figure 7:**
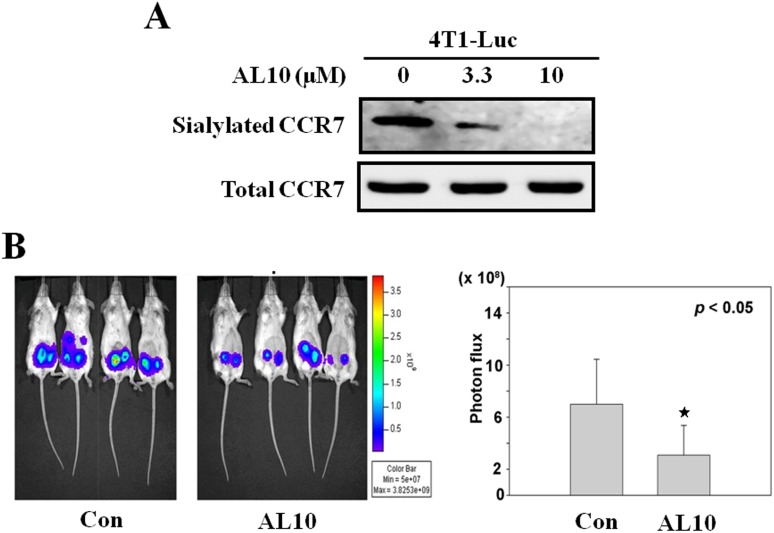
Inhibition of breast cancer growth by AL10 *in vivo*. (**A**) Highly metastatic murine 4T1-Luc breast cancer cells in which luciferase gene is constitutively expressed were treated with different doses of AL10 for 24 h and the sialylation status was investigated by lectin affinity precipitation and western blotting. (**B**) 4T1-Luc breast cancer cells (5×10^5^ cells/mice) were orthotopically injected into both mammary gland number four of female BALB/cByJNarl mice. Mice were randomly sorted into two groups. The treatment was initiated with either the vehicle alone (Control) or AL10 (3 mg/kg/day intraperitoneally into each mouse every day). *In vivo* photon emissions of 4T1-Luc cells in mammary glands were detected and photographed by IVIS 200 image system at day 10. Photon flux reading from mice were averaged and expressed as Mean±S.D. *p<0.05, when compared to the control group.

Finally, we tested the effect of AL10 on tumor growth *in vivo*. Although injection of human cancer cells into nude mice is generally conducted to assess the tumorigenicity, this model does not mimic *in vivo* situation because nude mice lack immunity. Therefore, we studied the effect of AL10 by using highly metastatic murine 4T1-Luc breast cancer cells in which luciferase gene is constitutively expressed. As shown in Fig. 7A, 4T1-Luc cells expressed high level of sialylated CCR7 which could be inhibited by AL10 dose-dependently. 4T1-Luc cells were orthotopically injected into both mammary gland number four of female BALB/cByJNarl mice. AL10 at the dose of 3 mg/kg/day significantly reduced tumor growth in mice (Fig. 7B). These data confirmed the anti-tumor activity of this sialyltransferase inhibitor *in vivo*.

## Discussion

During malignant transformation, tumor cells express highly sialylated molecules at their surface due to increased activity of the Golgi-localized STs [24], [25]. These highly sialylated molecules have been reported to be useful prognostic markers for cancer patients and are involved in cancer progression and metastasis. Zhou *et al* showed that CD133, a stem cell marker, could be sialylated in neural stem cells and glioma-initiating celles and sialylation regulated the protein stability of CD133 suggesting the control of stemness by protein sialylation [29]. By using mass spectrometry-based approach, a previous study demonstrated that a cell surface tumor antigen CD43 is sialylated *in vivo* and is highly expressed in colon and breast cancer. An UN1 monoclonal antibody which recognized the sialylated CD43 may be useful for cancer detection [30]. More recently, increased sialylation of P-glycoprotein and multiple drug resistance-related protein has been suggested to play critical role in the development of chemoresistance in acute myeloid leukemia cells [31]. These studies clearly demonstrate the importance of the understanding of protein sialylation in cancer.

It is well known that CCR7-expressing cancer cells exhibit high lymphatic invasion ability due to the chemotactic effect of its ligands CCL19 and CCL21 which predominantly expressed in lymphatic endothelial cells [19]. However, the sialylation status of CCR7 has not been investigated. In addition, whether CCR7 sialylation involves in metastatic behaviors is also unknown. We provide the first evidence that CCR7 is a sialylated receptor and reduction of CCR7 sialylation by AL10 attenuates CCL19-stimulated downstream signaling. We also performed multiple functional assays to assess the effect of AL10 on metastatic behaviors. Cell invasion, which is required for cancer cells to move across physiological barrier, and anti-anoikis, which is essential for cancer cells to survive in circulation system, is significantly enhanced by CCL19 stimulation. Importantly, CCL19-induced invasion and anti-anoikis are dramatically attenuated by AL10. The anti-cancer activity of AL10 in the inhibition of breast cancer in BALB/cByJNarl mice was also confirmed in this study.

Another important finding of this study is the elucidation of the control of basal and CCL19-induced cyclin D1 expression by AL10. GSK-3β has been shown to phoshorylate T286 of cyclin D1 and induce its degradation via proteasome-dependent pathway. Conversely, phosphorylation of GSK3β at Ser9 by AKT kinase or at Ser389 by p38 mitogen-activated protein kinase (MAPK) could inactivate GSK3β and increase cyclin D1 protein level by reducing degradation [32]–[34]. Because AKT signaling was activated by CCL19 in MDA-MB-231 cells, it is suggested that CCL19 induces AKT activation to inhibit GSK3β activity and, simultaneously, up-regulates cyclin D1. However, we could not exclude the involvement of p38 MAPK in CCL19-increased cyclin D1 because CCL19 also stimulated p38 MAPK activity in MDA-MB-231 cells (Fig. S2A). Treatment of SB202190, a specific p38 MAPK inhibitor, disrupted p38 activation and abrogated CCL19-increased cyclin D1 expression in these cells (Fig. S2B).

Compared to CCL19-induced cyclin D1 expression, the mechanism by which AL10 inhibits basal cyclin D1 protein is more complicated. A previous study showed that free cyclin D1 (unbound to CDKs) is ubiquitinated *in vivo* and this ubiquitination is independent of GSK3β and T286 phosphorylation [33]. Therefore, we hypothesize that AL10 can promote the ubiquitination and proteolysis of free cyclin D1 via three potential mechanisms. First, AL10 may activate Mirk/Dyrk1b kinase which could phosphorylate cyclin D1 on T288 residue to trigger protein degradation [35]. Second, AL10 may up-regulate the expression of the F-box proteins SKP2, FBX4 or FBXW8 to enhance the formation and activity of the E3 ligases responsive for the ubiquitination of cyclin D1 [36]–[38]. Third, AL10 may modulate N-terminal modification of cyclin D1. Certain proteins undergo N-terminal ubiquitination in which the polyubiquitin chain is attached to the α-NH_2_ group of the N-terminus of a target protein. Feng et al. demonstrated previously that N-terminal modification partially stabilized cyclin D1 protein [39]. Further investigations are needed to explore AL10-mediated cyclin D1 proteolysis. Cyclin D1 is important for the development and progression of breast cancer [40], [41]. In addition, overexpression of cyclin D1 is strongly associated with tamoxifen resistance in breast cancer cells [42], [43]. A number of therapeutic drugs have been shown to trigger cyclin D1 degradation in vitro [36]. Results of this study point out AL10 as another cyclin D1-degrading agent for breast cancer treatment. More importantly, AL10 can also inhibit sialyltransferases to suppress tumor invasion and metastasis.

In conclusion, we demonstrate for the first time that CCR7 is a sialylated receptor *in vivo* and verify the importance of sialylation on CCR7 function. In addition, we identify a novel agent AL10 as a dual therapeutic drug by inhibiting sialyltransferase activity and cyclin D1 expression.

## Supporting Information

Figure S1 **CCL21 did not significantly activate ERK and AKT signaling pathways in MDA-MB-231 cells.** Cells were treated with AL10 (10 µM) for 24 h and then stimulated with CCL19 (200 ng/mL) for 20 min. Total ERK, Total AKT, phospho-ERK and phospho-AKT were detected by Western blot analysis.
(TIF)

Figure S2 **p38 signaling pathway may be involved in the increase of cyclin D1 by CCL19.** (A) MDA-MB-231 cells were pre-treated with sialidase (200 mU) for 3 h and incubated with CCL19 (200 ng/mL) for 20 min. Total and phospho-p38 level was detected by Western blot analysis. (B) MDA-MB-231 cells were pre-treated with p38 inhibitor SB202190 for 2 h and then stimulated with CCL19 (200 ng/mL) for 20 min. Cyclin D1 expression and p38 activation were investigated.
(TIF)
