# Supplementary figures and images for: Inhibition of Chemokine (C-C Motif) Receptor 7 Sialylation Suppresses CCL19-Stimulated Proliferation, Invasion and Anti-Anoikis
Source: PLoS One. 2014 Jun 10;9(6):e98823. doi: 10.1371/journal.pone.0098823 (PMC4051673; doi:10.1371/journal.pone.0098823)

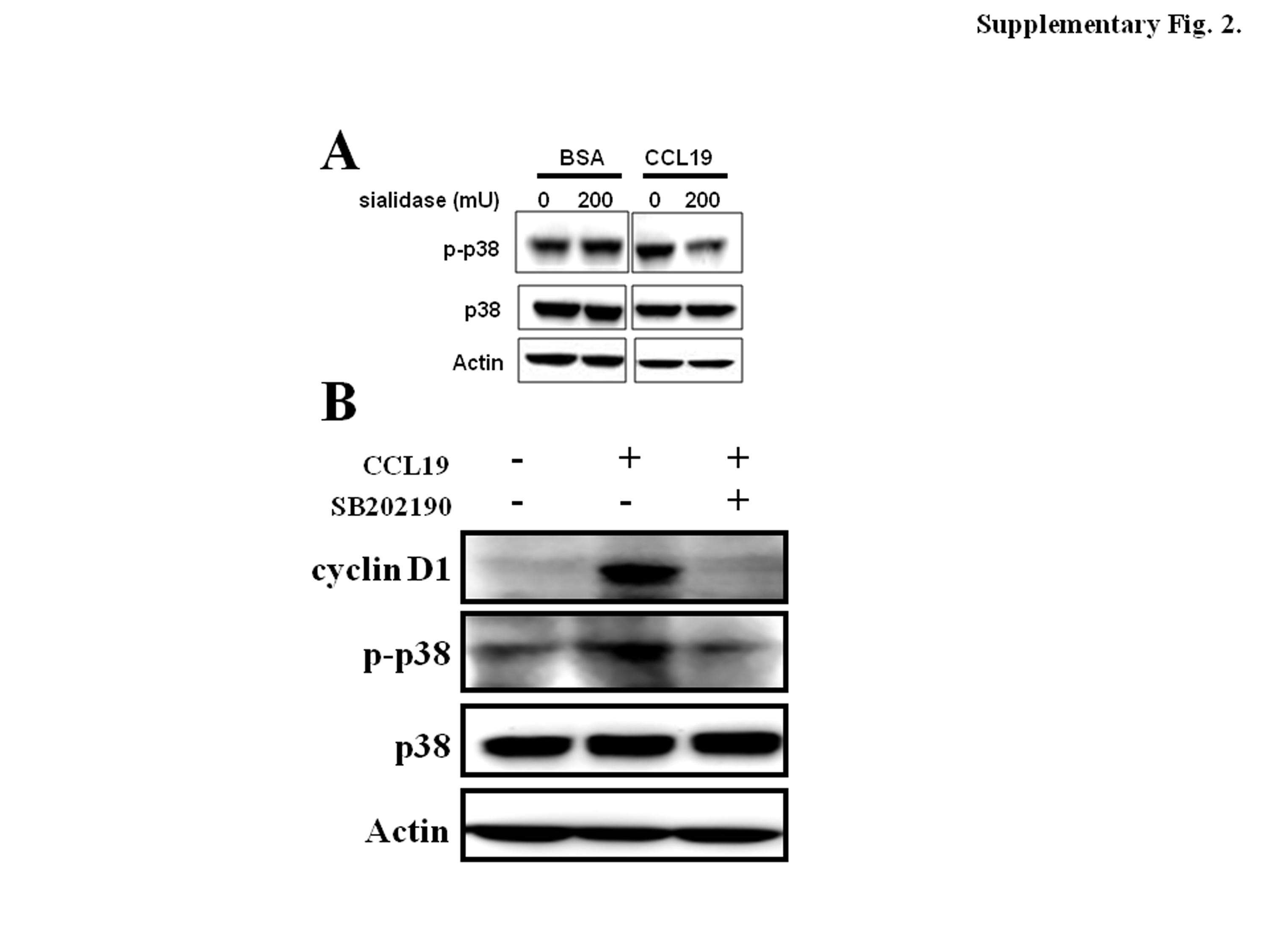

Supplement: Figure S2 — p38 signaling pathway may be involved in the increase of cyclin D1 by CCL19. (A) MDA-MB-231 cells were pre-treated with sialidase (200 mU) for 3 h and incubated with CCL19 (200 ng/mL) for 20 min. Total and phospho-p38 level was detected by Western blot analysis. (B) MDA-MB-231 cells were pre-treated with p38 inhibitor SB202190 for 2 h and then stimulated with CCL19 (200 ng/mL) for 20 min. Cyclin D1 expression and p38 activation were investigated. (TIF) [file pone.0098823.s002.tif]
